# Use of VacA as a Vaccine Antigen

**DOI:** 10.3390/toxins8060181

**Published:** 2016-06-08

**Authors:** Mati Moyat, Dominique Velin

**Affiliations:** Service of Gastroenterology and Hepatology, Department of Medicine, Lausanne University Hospital, CLE D203, CHUV, 155 Chemin des Boveresses, CH-1066 Epalinges, Switzerland; mati.moyat@epfl.ch

**Keywords:** VacA, vaccine, *H. pylori*

## Abstract

One of the major toxins secreted by *H. pylori* is the Vacuolating cytotoxin A (VacA) named after its ability to induce the formation of “vacuole”-like membrane vesicles in the cytoplasm of gastric cells. VacA has been associated with the disruption of mitochondrial functions, stimulation of apoptosis, blockade of T cell proliferation and promotion of regulatory T cells, thereby making it a promising vaccine target. Immunity to bacterial virulence factors is well known to protect humans against bacterial infections; hence, detoxified VacA has been evaluated as a vaccine antigen. Our short review summarizes the pre-clinical and clinical data that have been published on the use of VacA in the development of the *H. pylori* vaccine.

## 1. Introduction

*Helicobacter pylori* infection is one of the most common infections in human beings worldwide [1]. After entering the stomach, *H. pylori* colonizes the mucus gastric layer [2] but does not traverse the epithelial barrier [3], and therefore it is considered a non-invasive bacterium. Most of *H. pylori* organisms are free living in the mucus layer, but some organisms attach to gastric epithelial cells [3] and small numbers have been shown to invade those cells [4].

To survive into the stomach, *H. pylori* uses urease and α-carbonic anhydrase to generate ammonia and HCO_3_^2−^ enabling it to colonize and function at low pH conditions [5,6]. Furthermore, thanks to its flagella and shape, *H. pylori* penetrates the mucus layer [7]. Once established in the inner mucus layer, several outer membrane proteins such as BabA, SabA, AlpA, AlpB and HopZ mediate its adherence to gastric epithelial cells. After attachment to epithelial cells, bacterial effector molecules, both secreted (vacuolating cytotoxin (VacA) and cytotoxin-associated gene A (CagA)) or attached (components of the type IV secretion system (CagL)), modulate gastric epithelial cell behavior leading to loss of cell polarity, release of nutrients and chemokines (e.g., IL-8), and regulation of acid secretion via control of gastrin and H^+^/K^+^ ATPase [8,9].

Depending on *H. pylori* virulence factors, environmental factors and the host immunological status to bacterial infection, *H. pylori* infection can lead to several clinical complications such as gastritis, peptic ulcer disease, gastric cancer and mucosa-associated lymphoid tissue (MALT) lymphoma [10,11,12]. Hence, gastroenterologists use a combination of anti-secretory and antimicrobial agents to eradicate *H. pylori* [13,14]. However, like other antimicrobial treatments, the therapy may select resistant *H. pylori* strains [13,14]. Therefore, alternative therapies such as vaccine development against *H. pylori*, have been evaluated [15].

## 2. Source of *H. pylori*-Derived Antigens

The first murine vaccine studies utilized *H. felis* bacterial lysates or chemically inactivated whole-cell bacteria delivered to mice orogastrically along with cholera toxin (CT) or *E. coli* heat-labile enterotoxin (LT) as mucosal adjuvants [16]. However, the development of such vaccines would encounter quality control and regulatory issues related to the quality and reproducibility of vaccine preparations: therefore, defined antigens-based vaccines need to be developed.

Immunity to bacterial virulence factors is known to protect humans against bacterial infections. A well-known example is the use of detoxified pertussis toxin, filamentus hemagglutin and pertactin in the acellular pertussis vaccine to protect against *Bordetella pertussis* infections [16]. A similar strategy has been used to develop the *H. pylori* vaccine [17]. Indeed, as described above, *H. pylori* has a large arsenal of virulence factors that help the bacteria to colonize and persist within the stomach mucosa of its host. One of these virulence factors, Urease is essential for colonization and pathogenic events and due to its abundance has made it an ideal vaccine candidate [18]. In addition, proteomic analysis identified Urease as a dominant immunogenic protein [19]. Different routes of immunization (oral, intranasal, intrarectal, intramuscular) with urease (ureB subunit or inactive form of holoenzyme) associated with different adjuvants (CT, LT, alum, QS21) have been shown to protect or cure *H. felis* or *H. pylori* infections in mice and ferrets [17]. Although the first clinical trials evaluating Urease-based vaccines have been disappointing [18], Zeng and colleagues recently provided evidence that oral administration of *H. pylori* Urease B subunit fused to LT B subunit significantly reduced the acquisition of natural *H. pylori* infection in children [20]. While this study demonstrated the proof of concept for mucosal vaccine-induced protection against *H. pylori* infection, more work needs to be done in order to enhance its protective capacity. Indeed, whereas the vaccine protected more than 70% of recipients 1-year post vaccination, only 56% were protected 2–3 post vaccination [20,21]. Therefore, the formulation of an *H. pylori* vaccine containing other virulence factors may be considered to enhance vaccine efficacy. VacA is one of the virulence factors that have been evaluated as vaccine antigen.

## 3. Biological Activity of VacA

VacA is a secreted protein that has been reported to induce vacuolization, membrane anion-selective channel and pore formation, disruption of endosomal and lysosomal activity, and apoptosis in target cells. Furthermore, it has also been shown to induce IL-8 production in monocytes derived cell line [22]. Finally, VacA has been reported to have immunomodulatory properties through its reduction of T and B cells activation and induction of regulatory T cells differentiation, which allows *H. pylori* to establish a chronic infection [23,24]. VacA and many other *H. pylori* derived virulence factors, including NAP (Neutrophil-activating protein), and CagA constitute a complex network to regulate chronic gastric injury and inflammation that lead to gastric carcinogenesis [25].

Nearly all strains of *H. pylori* express a form of VacA. However, polymorphism may affect VacA toxigenic and pathogenic activity [25,26,27]. It is known that several regions of *vacA* are important for VacA toxicity. The *vacA* signal region encodes the N terminus of the toxin and may be active (type s1) or encodes an *N*-terminal extension, which blocks activity (s2) [26]. The *vacA* mid region may bind to a wide range of cells, causing toxicity (type m1), or to a smaller range (type m2) of cells [28]. Importantly for the development of VacA-based vaccine, mature type s1-m1 and type s2-m2 VacA toxins are about 75% identical in amino acid sequences overall [26]. Rhead and colleagues described a third determinant of VacA toxicity, called the intermediate (i) region [29]. They showed that two allelic variants of this region (i1 and i2) exist. This region determines toxic activity, and most importantly, they showed a significant correlation between the i1 region and gastric cancer (GC) [29,30].

## 4. Detoxified VacA as Vaccine Antigen

VacA cytotoxicity causes acute gastric epithelial erosion and ulceration, which excludes the use of the active toxin in humans [31]. VacA toxicity was found to be extremely sensitive to formaldehyde treatment, so complete inactivation of the toxin was achievable without compromising antigenicity [32]. After the formaldehyde treatment, the toxin is still capable to bind HeLa cells but does not induce vacuolization nor cause any epithelial damage when administered into the stomach of mice [32]. Moreover, the formaldehyde treated toxin retained some degree of its immunogenicity and was capable of inducing the production of neutralizing antibodies, although the neutralizing titers were 3.5 fold lower than those induced by untreated toxin [32]. Additionally, whole recombinant VacA molecule expressed in *Escherichia coli* and purified by affinity chromatography is nontoxic [33] and is a very convenient source of vaccine antigen. Indeed, when comparing to the native oligomeric toxin purified from cultures of *H. pylori*, the *E. coli*-expressed recombinant VacA was inactive in a HeLa cell assay of vacuolization and failed at inducing high titers of neutralizing antibodies in rabbits. Hence, the neutralizing epitopes in the native toxin protein are likely to be conformational and may exist only when the protein folds correctly into its native structure [33].

## 5. Preclinical Studies of the VacA-Based Vaccine

Significant levels of protection were achieved when infected mice were therapeutically vaccinated intragastrically with the non-toxic recombinant VacA combined with the non-toxic mutant of LT, LTK63. Importantly, therapeutic vaccination not only successfully eradicates *H. pylori* infection but also prevents a subsequent reinfection in the majority of vaccinated animals [34]. The results of prophylactic vaccination in animal models were also encouraging. Immunization with recombinant VacA, together with mucosal adjuvants, confers protection in mice [35]. Although vaccine-induced protection is not dependent on neutralizing antibodies [33], the whole VacA molecule was required to achieve protection. Indeed, the two VacA fragments TOX58 or TOX37 individually expressed, purified and used as antigen were unable to elicit significant protection against experimental *H. pylori* challenge [35]. An interesting alternative approach to the use of the whole VacA molecule has been published by Liu, K.Y. *et al*. The authors fused a fragment of VacA (aa 744–805) that contains CD4^+^ T cell epitopes to CagA and urease B fragments and expressed the fusion protein into an attenuated *Salmonella* vector [36]. Oral therapeutic immunization with this attenuated *Salmonella* expressing the CagA-VacA-UreB fusion protein, significantly decreased *H. pylori* colonization [36].

## 6. Anti-VacA Immune Response in Infected Individuals

Anti-VacA antibodies are present in serum and gastric juice from the majority of *H. pylori*-infected individuals [37] and neutralizing IgG anti-VacA antibodies are sometimes detectable in humans [38]. In addition, it has been shown that CD4^+^ lymphocytes isolated from the gastric epithelium of *H. pylori*-infected individuals proliferate in an antigen-dependent manner in the presence of VacA [39,40]. Although T and B cell responses directed toward VacA are present in infected hosts, the immune response is not sufficient to eradicate the established *H. pylori* infection. However, the detected immune responses directed toward VacA in chronically infected people demonstrate that VacA is immunogenic in the human population and might be a good candidate as vaccine antigen.

## 7. Impact of VacA Diversity on Vaccine-Induced Protection

Immunization with purified type s1-m1 VacA conferred protection against subsequent challenge with two *H. pylori* strains that produced vacuolating toxin activity *in vitro*. However, it failed to protect against challenge with a wild-type Tox^−^ strain that lacked detectable toxic activity for HeLa cells [35,41]. It is now known that many *H. pylori* strains that lack toxic activity (Tox^−^) for HeLa cells contain type s1-m2 or s2-m2 *vacA* alleles [26,35,41]. Based on these results, it can be speculated that the failure of an s1-m1 VacA antigen to induce protective immunity against a Tox^−^ strain may be due to antigenic diversity among different VacA proteins. In particular, it is possible that T epitopes present in the type s1-m1 VacA fail to cross-react with those present in the type s2-m2 VacA. Therefore, if VacA is to be used as a vaccine antigen, it might be appropriate to immunize with pooled VacA antigens derived from several different VacA families and/or include additional virulence factors of *H. pylori* [17].

## 8. VacA-Based Vaccine in Human Trials

Malfertheiner P *et al.* evaluated a multicomponent vaccine composed of purified recombinant VacA, CagA and NAP in a human phase I clinical trial [42]. Previous preclinical data clearly showed that VacA, CagA, and NAP cocktail given intramuscularly with aluminum hydroxide as adjuvant protected dogs against a challenge with *H. pylori* [42] and eradicated experimental infection [43]. The vaccine was next injected intra-muscularly to healthy, *H. pylori*-negative human volunteers [42]. The vaccine was well tolerated and elicited a long lasting serum antibody response in many subjects against VacA. *In vitro*, VacA restimulation of peripheral blood mononuclear cells induced cellular proliferation, which was paralleled by production of IFN-γ, suggesting that the vaccine had induced antigen-specific T-cell responses. Remarkably, a booster immunization 18–24 months after primary immunization elicited a strong anamnestic antibody response to VacA in all subjects, and VacA specific production of IFN-γ can be detected after *in vitro* VacA stimulation of peripheral blood mononuclear cells. These results suggested that the vaccine induced long-lasting immunity and immunologic memory in the B and T cell populations [42]. Although the results of this clinical trial were clearly positive and established that VacA is immunogenic and safe, to our knowledge this vaccine candidate did not progress to phase II study.

## 9. The Vaccine-Induced Protective Immune Responses

The demonstration that VacA injected to healthy individuals induces an anamnestic T cells response is of particular interest [42]. Indeed, preclinical studies clearly established the necessity to induce a *Helicobacter* specific CD4^+^ T cell response associated with an inflammatory reaction to reduce *Helicobacter* infection [44]. Since, T-cell responses directed against VacA have been detected in individuals chronically infected with *H. pylori* [39,40] or in heathly individuals injected intramuscularly with VacA [42], it can be speculated that VacA specific Th1, Th2 and/or Th17 cells may play critical roles in the vaccine-induced protection. These activated VacA specific CD4^+^ T cells will secrete a large set of cytokines such as IFN-γ, IL-17 and TNFα that promote the recruitment and the activation of inflammatory cells such as neutrophils [45], inflammatory monocytes [46] or mast cells [47] which participate to the reduction of bacterial load. In addition, our group recently provided evidence that Th17 cells infiltrating the gastric mucosa of vaccinated mice produce IL-22 and that IL-22-induced antimicrobial peptides are key determinants of vaccine-induced protection against *H. pylori* in mice [48]. In respect with the clinical development of future *H. pylori* vaccine, these pre-clinical data suggest that it might be of high interest to correlate the presence of circulating and/or stomach VacA-specific memory CD4^+^ T cells with the vaccine efficacy: hence, providing an immune correlate of vaccine protection.

## 10. Conclusions

Although heterogeneity exists in the expression of VacA in *H. pylori* isolates, limiting its potential as vaccine candidate if used alone, pre-clinical and clinical data clearly demonstrate the good potential of the molecule as vaccine antigen. However, since VacA is a protein that modulates many biological processes of gastric epithelial cells and immune cells [23,24,25], a complete evaluation of safety profile of VacA has to be performed before including recombinant VacA in a vaccine preparation.

## Abbreviations

The following abbreviations are used in this manuscript:

VacA: vacuolating cytotoxin
CagA: cytotoxin-associated gene A
LT: heat-labile enterotoxin
NAP: Neutrophil-activating protein
